# Clinical benefit of pembrolizumab in treatment of first line non-small cell lung cancer: a systematic review and meta-analysis of clinical characteristics

**DOI:** 10.1186/s12885-023-10959-3

**Published:** 2023-05-19

**Authors:** Wenjie Liu, Gengwei Huo, Peng Chen

**Affiliations:** Department of Thoracic Oncology, Tianjin Medical University Cancer Institute and Hospital, National Clinical Research Center for Cancer, Key Laboratory of Cancer Prevention and Therapy of Tianjin, Tianjin’s Clinical Research Center for Cancer, Tianjin, 300060 China

**Keywords:** Pembrolizumab, Non-small cell lung cancer, Predictor, Meta analysis

## Abstract

**Objective:**

Pembrolizumab has become an integral first line therapeutic agent for non-small cell lung cancer (NSCLC), but its potential predictive role in clinical and molecular characteristics remains to be clarified. Accordingly, we performed a systematic review and meta-analysis to evaluate the clinical benefit of pembrolizumab in treatment of first line NSCLC and to select individuals with the greatest potential benefit from pembrolizumab therapy, in order to obtain a more accurate treatment of NSCLC in immunotherapy.

**Methods:**

Mainstream oncology datasets and conferences were searched for randomized clinical trials (RCTs) published before August 2022. RCTs involved individuals with first line NSCLC treated with pembrolizumab monotherapy or in combination with chemotherapy. Two authors independently selected the studies, extracted data, and assessed the risk of bias. The basic characteristics of the included studies were recorded, along with 95 percent confidence intervals (CI) and hazard ratios (HR) for all patients and subgroups. The primary endpoint was overall survival (OS), and secondary endpoints was progression-free survival (PFS). Pooled treatment data were estimated using the inverse variance-weighted method.

**Results:**

Five RCTs involving 2,877 individuals were included in the study. Pembrolizumab-based therapy significantly improved OS (HR 0.66; CI 95%, 0.55–0.79; *p* < 0.00001) and PFS (HR 0.60; CI 95%, 0.40–0.91; *p* = 0.02) compared with chemotherapy. OS was substantially enhanced in individuals aged < 65 years (HR 0.59; CI 95%, 0.42–0.82; *p* = 0.002), males (HR 0.74; CI 95%, 0.65–0.83; *p* < 0.00001), with a smoking history (HR 0.65; CI 95%, 0.52–0.82; *p* = 0.0003), with PD-L1 tumor proportion score (TPS) < 1% (HR 0.55; CI 95%, 0.41–0.73; *p* < 0.0001) and TPS ≥ 50% (HR 0.66; CI 95%, 0.56–0.76; *p* < 0.00001), but not in individuals aged ≥ 75 years (HR 0.82; CI 95%, 0.56–1.21; *p* = 0.32), females (HR 0.57; CI 95%, 0.31–1.06; *p* = 0.08), never smokers (HR 0.57; CI 95%, 0.18–1.80; *p* = 0.34), or with TPS 1–49% (HR 0.72; CI 95%, 0.52–1.01; *p* = 0.06). Pembrolizumab significantly prolonged OS in NSCLC patients, regardless of histology type (squamous or non-squamous NSCLC), performance status (PS) (0 or 1), and brain metastatic status (all *p* < 0.05). Subgroup analysis revealed that pembrolizumab combined with chemotherapy had more favorable HR values than pembrolizumab monotherapy in improving the OS of individuals with different clinical and molecular features.

**Conclusion:**

Pembrolizumab-based therapy is a valuable option for first line treating advanced or metastatic NSCLC. Age, sex, smoking history and PD-L1 expression status can be used to predict the clinical benefit of pembrolizumab. Cautiousness was needed when using pembrolizumab in NSCLC patients aged ≥ 75 years, females, never smokers, or in patients with TPS 1–49%. Furthermore, pembrolizumab in combination with chemotherapy may be a more effective treatment regimen.

**Supplementary Information:**

The online version contains supplementary material available at 10.1186/s12885-023-10959-3.

## Introduction

Lung cancer has long been the principal cause of cancer morbidity and mortality worldly [1]. Approximately 85% of all lung cancers are non-small cell lung cancers (NSCLC) [2]. Immunotherapy based on immune checkpoint inhibitors (ICIs) has been an indispensable therapeutic method after surgery, radiotherapy, chemotherapy, and targeted therapy in the past 20 years and has been included in the 1st-line therapy for various malignancies [3–7]. The methods of immunotherapy for cancer include a variety of drugs recently developed to stimulate the immune system of patients and destroy tumor cells [8]. Among the many immune checkpoint pathways, the PD-1 receptor pathway is the most prominent in cancer treatment, through which anti-tumor immune activity is reduced. The PD-1 receptor exists on the surface of activated T cells surface [3, 9], which prevents tissue damage resulting from chronic inflammation and adjusts immune tolerance [10]. Programmed death ligands 1 and 2 (PD-L1 and PD-L2) interact with PD-1 to reduce T-cell receptor signal transduction and downregulate T-cell activation, proliferation, and T-cell-mediated anti-tumor response [11–13]. Pembrolizumab is a potent and highly selective IgG4-κ humanized anti-PD-1 monoclonal antibody that has been presently approved for the treatment of a variety of neoplasms, involving NSCLC, Hodgkin lymphoma, non-Hodgkin, melanoma, head and neck squamous cell carcinoma (HNSCC), urothelial cell and microsatellite instability (MSI) high cancer [14–16]. Pembrolizumab has a high affinity for the PD-1 receptor and a significant inhibitory effect on ligand interactivity and activity [17]. Results from several large clinical trials have shown the benefits of pembrolizumab for overall survival (OS) and progression-free survival (PFS) in advanced NSCLC [15, 18–25].

However, only a subset of patients with NSCLC would benefit from pembrolizumab treatment, and it is important to identify which group of patients has a greater chance of benefit. In this way, the benefit population can be screened, and on the other hand, the additional toxic side effects caused by unnecessary drug use can be reduced [26, 27]. PD-L1 expression determined by immunohistochemistry is one of the most influential biomarkers for detecting the efficacy of pembrolizumab. However, the detection reagent and platform are difficult to unify, and the puncture tissue size of many advanced patients is small and limited, which may affect the application of combined positive scores (CPS) or tumor proportion scores (TPS). Therefore, diagnostic accuracy may be limited. Additionally, some trials have shown that PD-L1 expression does not accurately identify individuals who are sensitive to immunotherapy. ICIs may be beneficial to individuals with negative PD-L1 expression [28, 29], and their accuracy in predicting response to immunotherapy is not ideal. Another promising biomarker is tumor mutational burden (TMB), however, its predictive efficacy remains controversial [30].

Current treatment options for individuals with advanced NSCLC have not yet met medical needs, especially those with recurrent or metastatic diseases. Although pembrolizumab-based therapy may have a strong and lasting tumor response, on the one hand, due to the lack of reliable biomarkers to predict the prognosis of individuals, the further clinical application of pembrolizumab is still a major challenge; on the other hand, because pembrolizumab is associated with specific adverse events, efforts are being made to identify predictable biomarkers to select individuals who obtain the greatest potential benefits from immunotherapy treatment. We conducted this systematic review and meta-analysis to analyze the impact of different clinical and molecular characteristics on the clinical benefit of pembrolizumab first line treatment in individuals with NSCLC to determine the appropriate biomarkers and guide the choice of treatment. We have provided a follow-up article in light of the PRISMA Reporting Checklist.

## Methods

### Inclusion and exclusion criteria

Articles were selected according to the PICOs principle (participants, intervention, comparison, and outcomes). The inclusion criteria were as follows: (I) pembrolizumab monotherapy or combined with chemotherapy versus chemotherapy for first line NSCLC individuals, and (II) OS and/or PFS available for each clinical and molecular feature subgroup. The exclusion criteria were: (I) pembrolizumab combined with anti-CTLA-4, antiangiogenic, radiotherapy or other specific therapy, (II) survival data were incomplete, (III) the control group only took placebo, (IV) if multiple articles were reported on the same RCTs, we selected the most recent, comprehensive data and the longest follow up, (V) if different articles in the same RCT looked at different subgroups, we brought into them all.

### Literature searching and data collecting

We searched for the target literature through PubMed, Embase Science Direct, Google Scholar, and the Cochrane library, as well as the minutes of main oncology meetings. The primary search terms were NSCLC, pembrolizumab, and a randomized controlled trial, supplemented by other terms. The literature was published prior to August 2022. The authors (WJL and GWH) independently selected the literature and extracted data from those studies. The third author (PC) resolved these differences. The title, first author, year of publication, study phase, line of therapy, study design, blinding, and survival outcomes were recorded.

### Quality assessment and statistical analyses

Quality assessment was independently evaluated by two authors (WJL and GWH) using the Cochrane bias tool. The primary endpoint was OS, and secondary endpoints was PFS. The chi-square test and *I*^*2*^ statistics were used to assess heterogeneity. Both *I*^*2*^ < 50% and *p* > 0.10 was regarded as heterogeneity-acceptable, employing the fixed-effect model; otherwise, the random-effect model was employed. Aggregated assessments, together with confidence intervals (CI) 95% and hazard ratios (HR) for all patients and subgroups were displayed using forest plots. Review Manager 5.3 was used for data statistics. Statistical significance was set at *p* < 0.05. The literature was excluded individually for sensitivity analyses.

## Results

### Study selection and characteristics

**Fig. 1:**
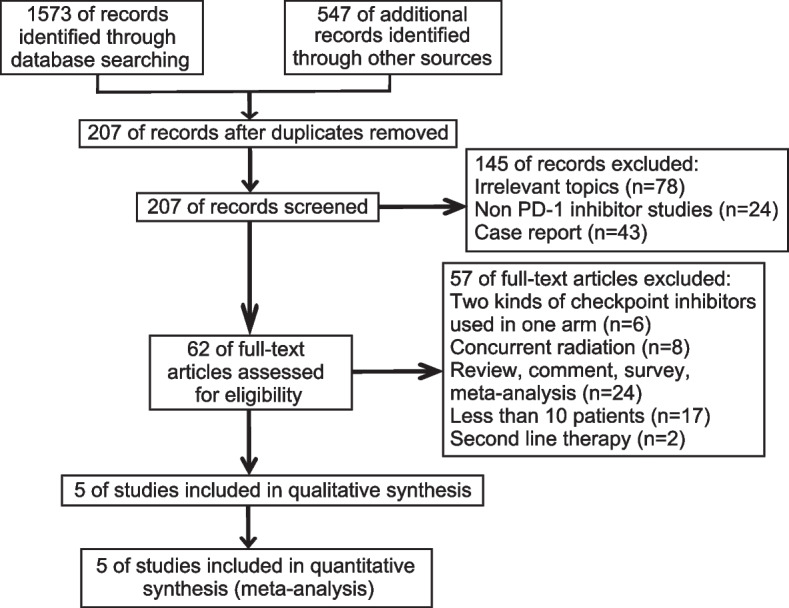
PRISMA flow diagram

**Fig. 2:**
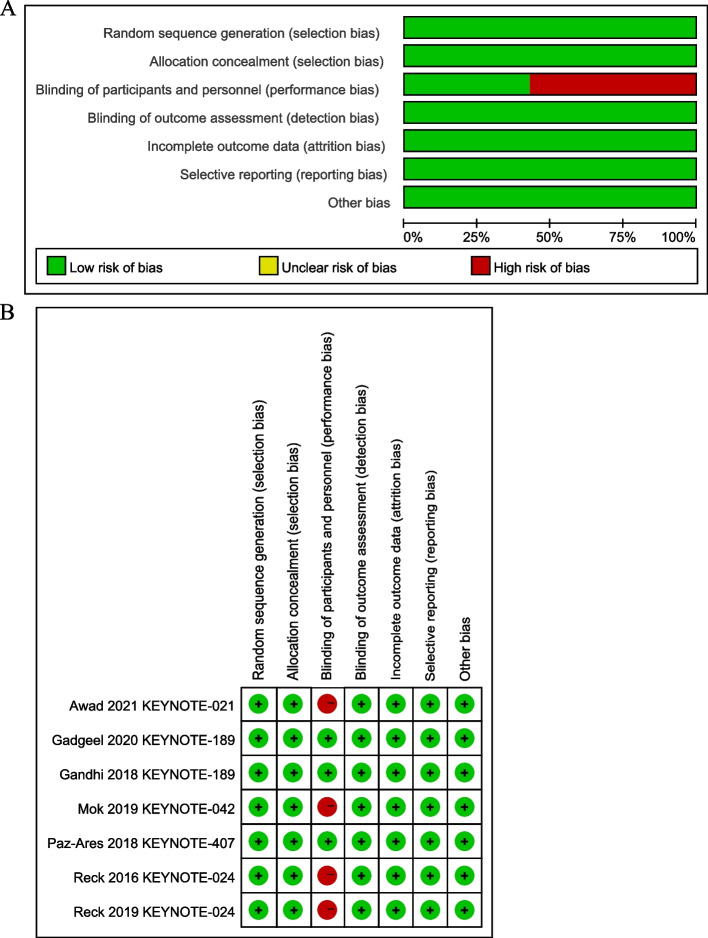
Assessment of bias risk, **A** risk of bias graph, **B** risk of bias summary

In total, 2,120 articles were obtained from the database. Figure 1 illustrated the filtering process. After filtering, 2,115 articles were excluded. Ultimately, 2,877 patients from five RCTs were included in our meta-analysis (Table 1). The included RCTs included one phase II [25], and four phase III RCTs [15, 19–23], published between 2016 and 2021. All RCTs had a low risk of bias, according to the risk of bias analysis (Fig. 2).

**Table 1:** Basic characteristics of included studies

**Reference**	**Trial**	**Treatment line**	**Study phase**	**Stage**	**ICI used** **(n)**	**Control arm** **(n)**	**Median ** **Age **	**Males (%)**	**Squamous (%)**	**Non-squamous (%)**	**Never smokers (%)**	**Tumor PD-L1 expression**			**ECOG**		**Primary endpoint**
												** < 1% (%)**	** ≥ 1% (%)**	**Unknown (%)**	**0 (%)**	**1 (%)**	
Awad et al. 2021 [25]	KEYNOTE-021	1L	II	IIIB/IV	Pembrolizumab + pemetrexed + carboplatin (60)	Pemetrexed + carboplatin (63)	64.3 (37–80)	39	0	100	20	36	64	0	43	56	ORR
Gandhi et al. 2018 [19]	KEYNOTE-189	1L	III	metastatic	Pembrolizumab + pemetrexed + platinum (410)	Placebo + pemetrexed + platinum (206)	64.5 (34–84)	58.9	0	100	11.9	30.8	63.0	6.2	43.2	56.0	OS and PFS
Gadgeel et al. 2020 [23]																	
Mok et al. 2019 [21]	KEYNOTE-042	1L	III	Advanced/metastatic	Pembrolizumab (637)	Platinum-based chemotherapy (637)	63 (57–69)	70.8	38.6	61.4	22.1	0	100	0	30.6	69.4	OS
Paz-Ares et al. 2018 [20]	KEYNOTE-407	1L	III	IV	Pembrolizumab + paclitaxel/nab- paclitaxel + carboplatin (278)	Placebo + paclitaxel/nab-paclitaxel + carboplatin (281)	65 (29–88)	81.4	97.7	2.3	7.3	34.7	63.2	2.1	29.2	70.8	OS and PFS
Reck et al. 2016 [15]	KEYNOTE-024	1L	III	IV	Pembrolizumab (154)	Platinum-based chemotherapy (151)	65.2 (33–90)	61.3	18.4	81.6	7.9	0	100	0	35.1	64.6	PFS
Reck et al. 2019 [22]

### Effects of pembrolizumab in NSCLC

**Fig. 3:**
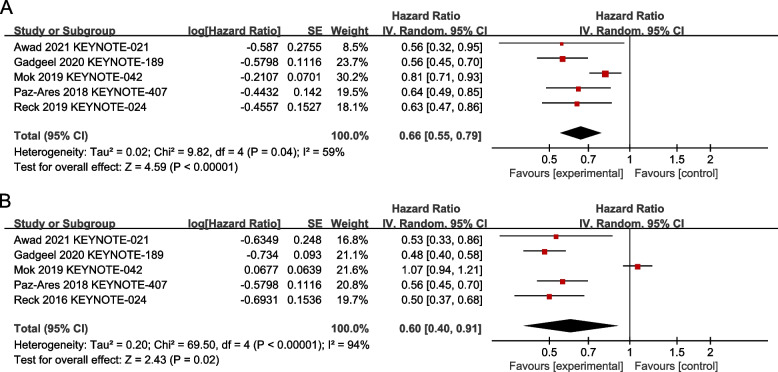
Forest plots of HRs comparing **A** OS and **B** PFS between pembrolizumab-based therapy and chemotherapy

Our meta-analysis revealed that pembrolizumab-based first line therapy significantly improved patients’ OS (HR 0.66; CI 95%, 0.55–0.79; *p* < 0.00001) (Fig. 3A) and PFS (HR 0.60; CI 95%, 0.40–0.91; *p* = 0.02) (Fig. 3B) compared with chemotherapy in five studies, respectively.

### Effects of pembrolizumab by age group

OS was significantly improved by pembrolizumab-based first line therapy versus chemotherapy in individuals aged < 65 years (HR 0.59; CI 95%, 0.42–0.82; *p* = 0.002) and ≥ 65 years (HR 0.68; CI 95%, 0.54–0.85; *p* = 0.0008). Surprisingly, we found no OS benefit in patients aged ≥ 75 years (HR 0.82; CI 95%, 0.56–1.21; *p* = 0.32) (Fig. S1A). Subgroup analysis revealed that the regimen of treatment did not affect OS improvement in individuals aged < 65 or ≥ 65 years (Table 2). In terms of PFS data from four studies, pembrolizumab significantly enhanced PFS versus chemotherapy in patients aged < 65 years (HR 0.48; CI 95%, 0.40–0.58; *p* < 0.00001) and ≥ 65 years (HR 0.63; CI 95%, 0.52–0.76; *p* < 0.00001) (Figure S2A and Table S1). No PFS data were available for the analysis of patients aged ≥ 75 years.

**Table 2:** Analyses of OS in subgroups of patients with varying clinical characteristics

Population	Subgroup	No. of studies	Test of association			Test of heterogeneity	
			HR	CI 95%	*p* value	*I*^*2*^	*p* value
Aged < 65 years	Total	4	0.59	0.42–0.82	0.002	74%	0.009
	monotherapy	2	0.78	0.65–0.93	0.005	24%	0.25
	combination therapy	2	0.47	0.36–0.61	< 0.00001	0%	0.51
Aged ≥ 65 years	Total	3	0.68	0.54–0.85	0.0008	0%	0.84
	monotherapy	1	0.64	0.42–0.98	0.04		
	combination therapy	2	0.69	0.53–0.91	0.007	0%	0.60
Aged ≥ 75 years	Total	2	0.82	0.56–1.21	0.32	10%	0.29
	monotherapy	2	0.82	0.56–1.21	0.32	10%	0.29
Male	Total	4	0.74	0.65–0.83	< 0.00001	21%	0.28
	monotherapy	2	0.68	0.46–1.00	0.05	71%	0.06
	combination therapy	2	0.70	0.56–0.88	0.002	0%	0.95
Female	Total	4	0.57	0.31–1.06	0.08	87%	< 0.0001
	monotherapy	2	0.90	0.71–1.15	0.41	0%	0.83
	combination therapy	2	0.32	0.23–0.46	< 0.00001	0%	0.34
Squamous	Total	3	0.71	0.60–0.83	< 0.0001	0%	0.71
	monotherapy	2	0.74	0.61–0.92	0.005	0%	0.94
	combination therapy	1	0.64	0.49–0.85	0.002		
Non-squamous	Total	4	0.68	0.52–0.87	0.002	70%	0.02
	monotherapy	2	0.73	0.50–1.07	0.10	73%	0.05
	combination therapy	2	0.59	0.48–0.72	< 0.00001	0%	0.36
PS 0	Total	4	0.67	0.54–0.83	0.0002	38%	0.18
	monotherapy	2	0.78	0.61–1.01	0.06	0%	0.97
	combination therapy	2	0.48	0.33–0.69	0.0001	0%	0.63
PS 1	Total	4	0.66	0.52–0.83	0.0005	66%	0.03
	monotherapy	2	0.71	0.48–1.04	0.08	73%	0.05
	combination therapy	2	0.59	0.47–0.74	< 0.00001	0%	0.36
Active or previous smoker	Total	3	0.65	0.52–0.82	0.0003	51%	0.13
	monotherapy	2	0.72	0.59–0.88	0.002	24%	0.25
	combination therapy	1	0.54	0.41–0.71	< 0.0001		
Never smoker	Total	3	0.57	0.18–1.80	0.34	80%	0.007
	monotherapy	2	1.00	0.73–1.36	0.99	0%	0.92
	combination therapy	1	0.23	0.10–0.54	0.0007		
With brain metastasis	Total	2	0.44	0.27–0.70	0.0006	0%	0.40
	monotherapy	1	0.73	0.20–2.62	0.63		
	combination therapy	1	0.41	0.24–0.67	0.0005		
Without brain metastasis	Total	2	0.60	0.50–0.73	< 0.00001	0%	0.70
	monotherapy	1	0.64	0.46–0.88	0.006		
	combination therapy	1	0.59	0.46–0.75	< 0.0001		
PD-L1 tumor proportion score < 1%	Total	2	0.55	0.41–0.73	< 0.0001	0%	0.58
	combination therapy	2	0.55	0.41–0.73	< 0.0001	0%	0.58
PD-L1 tumor proportion score ≥ 1%	Total	3	0.71	0.58–0.87	0.001	53%	0.12
	monotherapy	1	0.81	0.71–0.93	0.003		
	combination therapy	2	0.62	0.50–0.77	< 0.0001	0%	0.78
PD-L1 tumor proportion score 1–49%	Total	3	0.72	0.52–1.01	0.06	67%	0.05
	monotherapy	1	0.92	0.77–1.11	0.40		
	combination therapy	2	0.60	0.44–0.81	0.0007	0%	0.77
PD-L1 tumor proportion score ≥ 50%	Total	4	0.66	0.56–0.76	< 0.00001	0%	0.90
	monotherapy	2	0.67	0.57–0.80	< 0.00001	0%	0.66
	combination therapy	2	0.60	0.44–0.84	0.002	0%	0.81

### Effects of pembrolizumab by gender

Pembrolizumab significantly enhanced OS for the first line treatment in male (HR 0.74; CI 95%, 0.65–0.83; *p* < 0.00001), but not in female individuals (HR 0.57; CI 95%, 0.31–1.06; *p* = 0.08) compared with chemotherapy (Fig. S1B). Subgroup analysis showed that pembrolizumab as combination therapy (HR 0.70; CI 95%, 0.56–0.88; *p* = 0.002) improved the survival of individuals, but not as monotherapy in male individuals. In female individuals, subgroup analysis showed that pembrolizumab as combination therapy (HR 0.32; CI 95%, 0.23–0.46; *p* < 0.00001) improved the survival of individuals, but not as monotherapy (Table 2). PFS was substantially improved in both male (HR 0.55; CI 95%, 0.43–0.71; *p* < 0.00001) and in female individuals (HR 0.51; CI 95%, 0.35–0.74; *p* = 0.0004) (Figure S2B and Table S1).

### Effects of pembrolizumab by histomorphological subtypes

OS was significantly enhanced by pembrolizumab in first line therapy versus chemotherapy in both squamous (HR 0.71; CI 95%, 0.60–0.83; *p* < 0.0001) and non-squamous NSCLC (HR 0.68; CI 95%, 0.52–0.87; *p* = 0.002) (Fig. S1C). In non-squamous cell carcinoma, the analysis of subgroup by treatment regimen indicated that patients with pembrolizumab as combination therapy had a better survival (HR 0.59; CI 95%, 0.48–0.72; *p* < 0.00001), while patients treated with pembrolizumab as monotherapy had no substantial difference in survival compared with those who received chemotherapy. Subgroup analysis showed that both pembrolizumab monotherapy and combined chemotherapy prolonged the survival of individuals in squamous NSCLC (Table 2). PFS was also significantly improved in squamous (HR 0.54; CI 95%, 0.44–0.66; *p* < 0.00001) and non-squamous NSCLC individuals (HR 0.50; CI 95%, 0.43–0.58; *p* < 0.00001) (Figure S2C and Table S1).

### Effects of pembrolizumab by ECOG PS score

Pembrolizumab significantly enhanced OS versus chemotherapy in both individuals with performance status (PS) 0 (HR 0.67; CI 95%, 0.54–0.83; *p* = 0.0002) and PS 1 (HR 0.66; CI 95%, 0.52–0.83; *p* = 0.0005) (Fig. S1D). The analysis of the subgroup by the treatment regimen revealed that patients with PS 0 who received pembrolizumab as combination therapy had a better survival (HR 0.48;CI 95%, 0.33–0.69; *p* = 0.0001), but those who received monotherapy based on pembrolizumab did not. Subgroup analysis revealed that pembrolizumab in combination with chemotherapy prolonged the survival of individuals with PS 1 (HR 0.59;CI 95%, 0.47–0.74; *p* < 0.00001), but in monotherapy (Table 2). PFS was also substantially enhanced in patients for PS 0 (HR 0.47; CI 95%, 0.37–0.59; *p* < 0.00001) and PS 1 (HR 0.57; CI 95%, 0.49–0.67; *p* < 0.00001) (Figure S2D and Table S1).

### Effects of pembrolizumab by smoking status

Findings revealed that pembrolizumab-based 1st-line therapy provided a longer OS in individuals with a smoking history (HR 0.65; CI 95%, 0.52–0.82; *p* = 0.0003), but not in never smokers (HR 0.57; CI 95%, 0.18–1.80; *p* = 0.34) compared with chemotherapy (Fig. S1E). Patients with a smoking history had OS benefits regardless of the treatment regimen. In patients who had never smoked, pembrolizumab combined with chemotherapy improved survival (HR 0.23; CI 95%, 0.10–0.54; *P* = 0.0007), whereas monotherapy did not (Table 2).

### Effects of pembrolizumab by brain metastatic status

OS was significantly improved by pembrolizumab-based 1st-line therapy compared to chemotherapy in both individuals with brain metastases (HR 0.44; CI 95%, 0.27–0.70; *p* = 0.0006) and without brain metastases (HR 0.60; CI 95%, 0.50–0.73; *p* < 0.00001) (Fig. S1F). Subgroup analyses showed that only pembrolizumab combined with chemotherapy prolonged survival among patients with brain metastasis (HR 0.41; CI 95%, 0.24–0.67; *p* = 0.0005), but pembrolizumab monotherapy did not prolong survival. The treatment regimen did not affect OS improvement in individuals without brain metastasis (Table 2). Similarly, PFS was also substantially enhanced in patients with brain metastases (HR 0.44; CI 95%, 0.29–0.67; *p* = 0.0001) and without brain metastases (HR 0.49; CI 95%, 0.41–0.58; *p* < 0.00001) (Figure S2E and Table S1).

### Effects of pembrolizumab by PD-L1 tumor proportion score

OS was obviously prolonged by pembrolizumab for first line therapy compared with chemotherapy in individuals with PD-L1 TPS < 1% (HR 0.55; CI 95%, 0.41–0.73; *p* < 0.0001), TPS ≥ 1% (HR 0.71; CI 95%, 0.58–0.87; *p* = 0.001), and TPS ≥ 50% (HR 0.66; CI 95%, 0.56–0.76; *p* < 0.00001), while not in individuals with TPS 1–49% (HR 0.72; CI 95%, 0.52–1.01; *p* = 0.06) (Fig. S1G). Subgroup analysis revealed that for patients with TPS < 1%, 1st-line combined therapy had OS benefits, and there were no relevant data based on monotherapy. The analysis of subgroups by treatment regimen revealed that for patients with TPS 1–49%, the pooled HR involving three studies based on pembrolizumab combined chemotherapy was 0.60 (CI 95% 0.44–0.81; *p* = 0.0007). Only one research was referred to the monotherapy, with the HR of 0.92 (CI 95% 0.77–1.11; *p* = 0.40). Patients with TPS ≥ 1% and ≥ 50% had OS benefits regardless of the regimen of therapy (Table 2). PFS was significantly improved in patients with PD-L1 TPS < 1% (HR 0.66; CI 95%, 0.52–0.84; *p* = 0.0008), TPS 1–49% (HR 0.53; CI 95%, 0.42–0.69; *p* < 0.00001), and TPS ≥ 50% (HR 0.50; CI 95%, 0.32–0.76; *p* = 0.001) (Figure S2F and Table S1).

### Sensitivity analysis and publication bias

The literature was excluded individually for sensitivity analyses, revealing that the main outcomes after excluding this research did not differ substantially from past outcomes, indicating low sensitivity, credibility, and robustness of the outcomes (Table S2). These sensitivity analyses did not alter the prognostic factors in the entire cohort. Furthermore, no obvious publication bias was discovered based on the funnel plots of OS and PFS (Figure S3) and the funnel plots of OS and PFS for each subgroup (Figure S4 and S5).

## Discussion

The success of PD-1 inhibitors in the treatment of NSCLC was a vital milepost in the history of tumor treatment [31], which has been shown to have long-lasting anti-tumor efficacy and has significantly revolutionized the therapeutic paradigm of advanced/metastatic NSCLC [32–35]. Pembrolizumab strongly inhibits the PD-1/PD-L1 immune signaling pathway. This prospective neobiological drug has rightfully earned its place as one of the most successful new therapies for cancer. Better efficiency and treatment tolerance demonstrated that pembrolizumab was superior to chemotherapy [36–39]. Although a crucial breakthrough has been made, this persistence only reaches a few patients (about 20%) [40] and pembrolizumab was associated with specific adverse events, which highlights the urgency of biomarkers to predict the long-term clinical benefit of treatment. The expression of PD-L1 in tumor cells was related to clinical benefits and currently routinely serves as a biomarker in the clinical practice of NSCLC [36]. Nonetheless, PD-L1 remains a defective biomarker because a few high expression individuals are non-responsive, whereas PD-L1 negative or low expression individuals are usually observed to be responsive. In NSCLC, TMB was also related to PFS and objective response rate (ORR) with anti-PD-1 antibodies [41, 42]. The use of TMB in clinical practice demands constant efforts to reconcile quantitative calculation methods, solutions for the rapid return of results, costs, and intratumoral and inter-tumor heterogeneity. To date, the association of TMB with OS has either gone undetected or has been limited to relatively short followings [43, 44]. Studies on tumor specimens from melanoma patients have shown that the response to PD-1/L1 blockers was dependent on tumor infiltration of activated CD8^+^ T effector cells prior to treatment [45]. The impact of CD4^+^ T lymphocytes in the treatment of PD-1 inhibitors has not been well studied, and there was no clear correlation yet. Therefore, the predictive value of biomarkers such as CD4^+^, CD8^+^, PD-L1, and TMB for PFS and OS in patients with NSCLC receiving anti-PD-1 antibodies was limited. In addition, many biomarkers have been proposed to determine the therapeutic effect of pembrolizumab treatment, such as tumor size, albumin, lymphocyte count, and lactate dehydrogenase level [41, 46–49], however, none of these biomarkers accurately predicted the efficacy of pembrolizumab. The demand for a robust, non-invasive biomarker to predict the clinical benefit of pembrolizumab for first line treatment in patients with advanced NSCLC was still lacking. The development of reliable biomarkers to accurately identify individuals who could benefit from immunosuppressive agents is essential for optimizing the treatment of advanced NSCLC and selecting the most effective treatment to maximize clinical benefit.

A total of 2,877 patients from five RCTs were included in our meta-analysis. Our meta-analysis revealed that pembrolizumab was an effective treatment for NSCLC in first line. The pooled estimate for both OS and PFS was substantially improved compared with chemotherapy. Did pembrolizumab have a positive effect on patients with different clinical characteristics? We further searched for the dominant population using a subgroup analysis. To the best of our knowledge, our study was one of the largest meta-analyses to explore the efficacy of pembrolizumab in patients with NSCLC with different clinical features.

Age is a well-known hazard factor for carcinoma occurrence and progression [50], and is associated with poor prognosis [51, 52]. Similarly, if elderly patients benefit less from ICIs, then age related changes in the immune system may result in a decline in immune function, which has also triggered debate [53, 54]. Age was reported to be related to changes in the immune system [55, 56], which may alter cytokine dynamics [57] and reduce CD8^+^ T-cell proliferation [58]. This has also been associated with reduced T-cell function [59], CD28 expression [60, 61], and costimulatory signals for T-cell activation [62]. However, Elias et al. [63] discussed the clinical benefit and safety of PD-1/L1 inhibitors in patients with NSCLC, melanoma, and kidney carcinoma and observed no significant age-related differences in OS and side effects between older and younger individuals in clinical trials. This finding was consistent with the results of our study. Interestingly, in patients aged ≥ 75 years, pembrolizumab did not result in better OS than chemotherapy. This result was inconsistent with the results of a recently published study that found that among patients with NSCLC aged > 75 years, pembrolizumab plus chemotherapy achieved longer OS and PFS [64]. Comparing the results of our meta-analysis, we enrolled patients aged ≥ 75 years who were all treated with 1st-line pembrolizumab monotherapy, whereas in the study by Yang et al., all patients were treated with pembrolizumab plus chemotherapy. This may have contributed to the different results, but the study also points out that pembrolizumab plus chemotherapy leading to treatment discontinuation (26% vs. 5%) caused by adverse events was higher than that of chemotherapy. Therefore, pembrolizumab plus chemotherapy should be prioritized based on close monitoring of adverse reactions in patients aged ≥ 75 years. In addition, according to existing knowledge in this field, a substantial reduction in OS with ICIs was found in patients with cancer aged ≥ 75 years. This may be due to genetic changes in tumor cells and the activation of tumorigenic signals, which initiate inflammation, angiogenesis, or metabolic changes, eventually leading to immune resistance or evasion [65–67].

Differences in the immune systems of males and females may be associated with the natural process of chronic inflammatory diseases such as tumors [68, 69]. Gender differences in the efficacy of immunotherapy on PFS/OS were also explored, and the improvement in OS with pembrolizumab differed significantly between male and female patients with male patients appearing to benefit more from pembrolizumab treatment than women. This may be due to the fact that female tumors must avoid more effective immune surveillance mechanisms and undergo more intensive immune editing processes to metastasize [70]. This ability of female tumors to escape immune surveillance may reduce the immunogenicity of advanced female tumors, and the immune escape mechanism is stronger than that of similar tumors in males [71]. Therefore, these cells may be more resistant to immunotherapy. Second, a higher TMB was a powerful predictor of the benefits of ICIs [72]. TMB was significantly higher in male patients with multiple tissue types, including melanoma and NSCLC [73–75]. It is important to note that complex interactions among genes, hormones, environment, and symbiotic microbiome components may also affect the gender response to pembrolizumab-based treatment [76–80]. Furthermore, it has also been shown that EGFR mutant type NSCLC tumors are significantly less sensitive to ICIs than EGFR wild type NSCLC tumors, and are more common in female patients than in male patients [81].

The approval of pembrolizumab as an anti-PD-1 antibody in the 1st-line therapy for advanced/metastatic NSCLC with PD-L1 ≥ 50% (all histologies) provided direction for a new therapeutic option for patients with squamous cell carcinoma, accounting for approximately 25–30% of cases [82, 83]. Compared with non-squamous NSCLC, patients with squamous histological type are usually older at diagnosis and have advanced disease [84, 85], have a higher incidence of comorbidity [86, 87] and are more likely to invade larger vessels [88–90]. In addition, mutations approved for targeted therapy were rare in squamous NSCLC [91–95]. Therefore, treatment options for improving the prognosis of these patients were limited, especially the 1st-line options for advanced stages [96, 97]. In our analysis, pembrolizumab exhibited longer OS than chemotherapy in individuals with squamous and non-squamous NSCLC, suggesting that histology may not be a prognostic factor or predictor of clinical efficacy outcomes. Meanwhile, individuals with both squamous and non-squamous NSCLC benefit from 1st-line treatment, which provided an early treatment regimen [98]. It should be pointed out that although < 30% of individuals who meet the pembrolizumab therapy conditions may have enhanced survival, the present guidelines still recommend 1st-line platinum-based dual chemotherapy for the majority of treatment-naive individuals, even those with PS 0 and 1 [99–103]. For individuals with PS 2 or elderly patients, systemic treatment was recommended because of possible comorbidity and toxicity [96, 97], but there was evidence that individuals with PS 2 complicated with diseases or elderly patients usually did not receive chemotherapy [104–106]. The benefit of pembrolizumab observed in individuals with squamous NSCLC was significant and may promote better survival outcomes. Therefore, could pembrolizumab be a promising alternative to chemotherapy for these patients?

At present, the evaluation of PS should not be ignored as an important decision parameter when patients with advanced cancer choose treatment. The ECOG PS guidance for clinicians was a more reliable reflection of the real condition than biological age. This was widely supported in the literatures [107–110]. Meanwhile, a good PS (0 or 1) may play a vital role in guiding treatment decisions for NSCLC [111]. Previous studies have shown that earlier pembrolizumab treatment most likely confers the maximum survival benefit among individuals with PS of 0 and 1. Immunotherapy was also often the preferred option in practice for individuals with PS 0 and 1, who were not receiving 1st-line pembrolizumab therapy, and whose disease has progressed after 1st-line chemotherapy [112]. Most RCTs on pembrolizumab have included only patients with NSCLC with an ECOG PS of 0 or 1. We did not consider pembrolizumab as a treatment option for patients with PS ≥ 2, because the extent of the benefit was unclear. To achieve optimal use of pembrolizumab in clinical practice and improve patient outcomes, we investigated whether ECOG PS could be used as a potential biomarker to predict pembrolizumab clinical benefit. Our results indicated that early treatment with pembrolizumab improved OS in both individuals with PS 0 and 1, and that combined chemotherapy provided further OS benefits. Similar conclusions have been drawn for other immunotherapies such as atezolizumab and nivolumab [113, 114]. In addition, Ksienski et al. revealed that individuals with ECOG PS 2 or 3 had a higher rate of serious adverse effects after pembrolizumab treatment compared to patients with ECOG PS 0/1 [115]. Pembrolizumab should be carefully considered when considering treatment for patients with poor ECOG PS. To determine the definite efficacy of pembrolizumab versus chemotherapy, subsequent RCTs in individuals with an ECOG PS of 2 or 3 are required.

Smoking status was highly correlated with the incidence of NSCLC and has an obvious effect on the efficacy and tolerance of many lung cancer drugs [116]. In terms of immunotherapy, Li et al. [117] and Lee et al. [118] showed that smoking status affects the survival benefit of patients. We assessed the association between smoking status and survival benefits in NSCLC patients treated with pembrolizumab. Similar studies have been conducted in previous meta-analyses; however, there was a lack of detailed subgroups and multiple PD-1/PD-L1 inhibitors involved [117, 119, 120]. Our results show that compared with chemotherapy, the use of pembrolizumab significantly prolonged the survival of former and current smokers with NSCLC but did not improve the survival of patients who never smoked. This result was similar to that of Kim et al., who observed that ICIs had a substantially longer OS than chemotherapy in individuals with a history of smoking, but no benefit was found in individuals who had never smoked [121]. Other studies have shown that the ORR was substantially higher in non-squamous NSCLC individuals with a smoking history than in never-smokers (21.5% vs. 9.2%, *p* = 0.0001) when treated with nivolumab [122]. This may be because, firstly, smoking could significantly increase TMB [123], making tumors more immunogenic, thus increasing the anti-tumor effect of pembrolizumab [41]. Secondly, smokers and non-smokers have different molecular profiles of lung cancer as well as different tumor microenvironments [124], which may also influence the susceptibility of patients to pembrolizumab treatment. Thirdly, smoking promotes tumorigenesis by allowing pulmonary epithelial cells to evade adaptive immunity. The carcinogen benzoapyrene (BAP) can also induce PD-L1 expression in pulmonary epithelial cells in vitro and in vivo, and PD-1 inhibitors can significantly inhibit BAP-induced lung cancer [125]. However, further subgroup analysis found that in the pembrolizumab combined chemotherapy group, patients who never smoked also gained survival benefits. Thus, we believe that chemotherapy may have increased the efficacy of pembrolizumab in the combination therapy group, which requires further experimental confirmation. In summary, smoking status should be fully considered during treatment, and combined treatment may be more effective for patients with NSCLC who never smoke. In addition, studies have shown that recurrent molecular alterations were frequently detected in never-smokers, and activated T-cell therapy may be a possible strategy for treating patients with lung cancer [126, 127]. Therefore, the smoking status appears to be an appropriate biomarker.

NSCLC accounts for approximately 80% of all lung cancer cases, and nearly half of patients with NSCLC have distant metastases at their initial diagnosis. The brain is one of the most common metastatic sites [128]. Approximately one-third of individuals with advanced NSCLC have brain metastasis [129]. The prognosis of patients with NSCLC after the diagnosis of brain metastasis was consistently poor, with an estimated median OS of 7.8 months [130]. Previous RCTs of pembrolizumab administered to individuals with advanced NSCLC have reported good activity and some long-lasting system responses [36]. However, trials using these and other ICIs typically exclude patients with brain metastases. Our analysis specifically targeted pembrolizumab in individuals with untreated advanced NSCLC, in which patients with NSCLC with or without brain metastasis benefited from pembrolizumab treatment, underscoring the potential activity of pembrolizumab in individuals with central nervous system diseases. Further subgroup analysis showed that both pembrolizumab monotherapy and combined chemotherapy prolonged the OS in patients without brain metastases. Some studies have shown that individuals with brain metastases from NSCLC could benefit from immunotherapy either as monotherapy [15] or in combination with chemotherapy [19, 20]. However, we did not observe any benefit of pembrolizumab as a 1st-line monotherapy in NSCLC patients with brain metastasis, while individuals were observed to mainly benefit from pembrolizumab-based early combination therapy. This may be due to the disruption of the blood–brain barrier and the formation of new blood vessels that enable chemotherapy to pass through the brain [131]. In preclinical studies, chemotherapy drugs showed immune regulatory properties that could increase tumor immunogenicity [132–134]. Therefore, pembrolizumab combined with chemotherapy appears to be more effective in patients with brain metastasis. Moreover, our findings correspond with a real-world study that assessed the efficacy of pembrolizumab plus carboplatin and pemetrexed in individuals with advanced non-squamous NSCLC with or without brain metastasis, and the combined activity of the two groups was demonstrated [135].

PD-L1 is expressed in tumor cells and tumor-infiltrating immune cells [136]. The combination of PD-L1 with the PD-1 receptor on activated T-cells reduces the immune response of T cells and prevents tumor cell eradication [137, 138]. Besides playing a central role as a critical factor in current immunotherapy regimens, PD-L1 has also been demonstrated in several studies to emerge as a potential prognostic biomarker that can predict which individuals with NSCLC were more responsive to ICIs [139–144]. Our study specifically targeted the impact of PD-L1 expression in the treatment of pembrolizumab, similarly, suggesting that the expression status of PD-L1 appeared to be a significant biomarker for predicting pembrolizumab efficacy. Furthermore, we found that when treated with a single drug (individuals with PD-L1 TPS ≥ 50%) and combined chemotherapy (regardless of tumor cell PD-L1 expression) for advanced or metastatic NSCLC, pembrolizumab increased the OS compared to platinum-based chemotherapy. It should be noted that the application of pembrolizumab as a monotherapy to improve survival in individuals with negative PD-L1 expression is not recommended because of the lack of experimental results and data support. It could be seen that when TPS ≥ 50%, pembrolizumab showed a more stable survival improvement. Previous studies have shown that PD-L1 TPS ≥ 50% was related to a statistically substantial improvement in survival compared to individuals with lower PD-L1 expression in NSCLC [15, 145, 146]. For the 1st-line therapy of patients with metastatic NSCLC receiving pembrolizumab monotherapy, TPS ≥ 1% (preferably TPS ≥ 50%) was required to initiate treatment. PD-L1 was a continuous variable related to ICI; the higher the expression, the higher the possibility of a reaction. However, in some patients, the high expression itself was not enough to react with pembrolizumab, while in other patients, although the expression was very low, the reaction does occur. This may be explained by the multiple parameters that influence the anti-tumor immune response. Patients with some favorable parameters, such as normal LDH, normal CRP, and lower TMB (such as early stage disease), may have fewer immune responses influenced by PD-L1 expression [147]. In addition, factors such as the diversity of PD-L1 antibodies and platforms, diversity of tissue processing methods, heterogeneity of PD-L1 expression within the same cancer, and heterogeneity between primary cancer metastasis showed that PD-L1 expression was obviously imperfect as a biomarker for pembrolizumab treatment [148].

Our meta-analysis revealed that the OS benefit was not affected by histology type, ECOG PS score, or brain metastatic status. The PFS benefit was not affected by age, sex, histology type, ECOG PS score, or brain metastatic status. In addition, we found that pembrolizumab combination chemotherapy possessed a more beneficial HR value than pembrolizumab monotherapy in improving patient OS, and we recommend that pembrolizumab-based combination therapy is preferred for NSCLC in clinical applications.

Although our results provided some useful conclusions, we acknowledge the following limitations. Firstly, the data came from published articles with prearranged subgroups that were not obtained from the characteristics of the individual patients themselves. Therefore, some potential variants (such as TMB) were omitted from our analysis, which could lead to differences between our results on the clinical activity of pembrolizumab and the current results, which would cause some imprecision in the results and potential bias. Therefore, our subgroup analysis findings were still enlightening but were not conclusive. Secondly, some of the results with heterogeneous due to the fact that patients in a subgroup had diversity clinical and molecular characteristics, and the results about the biomarkers were somewhat scattered. For example, PD-L1 expression in tumors, which was distributed differently between the sexes, may influence pembrolizumab response. Thirdly, despite our comprehensive and systematic search in mainstream databases, the number of retrieved articles was still relatively small, and some subgroup analyses performed in this study included only a few trials. It could not be ruled out that insufficient statistical power may explain the results obtained from these subgroup analyses. Therefore, caution should be exercised when interpreting the results. Fourthly, we have not compared the clinical benefit of pembrolizumab monotherapy versus pembrolizumab combined with platinum-based chemotherapy. Further research is needed using the method of network meta-analysis. Fifthly, the majority of studies have demonstrated a rapid decline in PFS and OS in the pembrolizumab arm compared to chemotherapy, especially in the first six months following randomization, which may violate the proportional hazard assumption—indicating that during the early post-randomization phase, the HR may be higher than the given HR in the publication before becoming lower (which may be lower than the given HR in the publication) in subsequent phases.

Pembrolizumab, an immune drug, enhances the body's natural defenses against tumors. In the practical application of pembrolizumab, a comprehensive evaluation of these clinical predictors will help better direct the treatment of NSCLC individuals, shape clinical decision-making of NSCLC, and assist in the planning of future RCTs, in order to achieve personalized treatment and finally apply the principles of precision medicine. Beyond that, there is a need to continue investigating the potential benefit of pembrolizumab as part of a multi-modal treatment approach for advanced/metastatic NSCLC. In fact, we are beginning an exciting journey for patients and scientific research.

In summary, this meta-analysis revealed that pembrolizumab-based therapy is a valuable option for the treatment of advanced/metastatic NSCLC. Age, sex, smoking history and PD-L1 expression status can be used to predict the clinical benefit of pembrolizumab. Cautious use of pembrolizumab is needed in patients with NSCLC aged ≥ 75 years, females, never smokers, or in patients with TPS 1–49%. Furthermore, pembrolizumab combined with chemotherapy may be a more effective treatment option, regardless of the clinical and molecular characteristics of patients with NSCLC. The results of this analysis will contribute to the design of future clinical trials based on predefined subgroups.

## Supplementary Information

**Additional file 1.**

## Data Availability

All data generated or analysed during this study are included in this published article and its supplementary information files.
