# Supplementary material for: Clinical benefit of pembrolizumab in treatment of first line non-small cell lung cancer: a systematic review and meta-analysis of clinical characteristics
Source: BMC Cancer. 2023 May 19;23:458. doi: 10.1186/s12885-023-10959-3 (PMC10197372; doi:10.1186/s12885-023-10959-3)
Supplement: Supplementary file 1 — Additional file 1. [file 12885_2023_10959_MOESM1_ESM.pdf]

Table S1 Analyses of PFS in subgroups of patients with varying clinical characteristics

Population	Subgroup	No. of studies	Test of association			Test of heterogeneity	
			HR	CI 95%	*p* value	I^2	*p* value
Aged <65 years	Total	3	0.48	0.40-0.58	<0.00001	1%	0.36
	monotherapy	1	0.61	0.40-0.92	0.02		
	combination therapy	2	0.46	0.37-0.56	<0.00001	0%	0.43
Aged ≥65 years	Total	3	0.63	0.52-0.76	<0.00001	42%	0.18
	monotherapy	1	0.45	0.29-0.70	0.0004		
	combination therapy	2	0.68	0.55-0.84	0.0004	0%	0.42
Male	Total	3	0.55	0.43-0.71	<0.00001	57%	0.10
	monotherapy	1	0.39	0.26-0.58	<0.00001		
	combination therapy	2	0.61	0.51-0.73	<0.00001	0%	0.48
Female	Total	3	0.51	0.35-0.74	0.0004	57%	0.10
	monotherapy	1	0.75	0.46-1.21	0.23		
	combination therapy	2	0.42	0.32-0.55	<0.00001	0%	0.46
Squamous	Total	2	0.54	0.44-0.66	<0.00001	37%	0.21
	monotherapy	1	0.35	0.17-1.71	0.004		
	combination therapy	1	0.56	0.45-0.70	<0.00001		
Non-squamous	Total	3	0.50	0.43-0.58	<0.00001	0%	0.77
	monotherapy	1	0.55	0.39-0.76	0.0004		
	combination therapy	2	0.49	0.41-0.58	<0.00001	0%	0.64
PS 0	Total	3	0.47	0.37-0.59	<0.00001	0%	0.93
	monotherapy	1	0.45	0.26-0.77	0.004		
	combination therapy	2	0.47	0.36-0.61	<0.00001	0%	0.74
PS 1	Total	3	0.57	0.49-0.67	<0.00001	0%	0.68
	monotherapy	1	0.51	0.35-0.73	0.0003		
	combination therapy	2	0.59	0.49-0.70	<0.00001	0%	0.60
With brain metastasis	Total	2	0.44	0.29-0.67	0.0001	0%	0.64
	monotherapy	1	0.55	0.20-1.56	0.26		
	combination therapy	1	0.42	0.27-0.67	0.0002		
Without brain metastasis	Total	2	0.49	0.41-0.58	<0.00001	0%	0.85
	monotherapy	1	0.50	0.36-0.68	<0.0001		
	combination therapy	1	0.48	0.39-0.59	<0.00001		
PD-L1 tumor proportion score <1%	Total	2	0.66	0.52-0.84	0.0008	0%	0.84
	combination therapy	2	0.66	0.52-0.84	0.0008	0%	0.84
PD-L1 tumor proportion score ≥1%	Total	3	0.61	0.31-1.18	0.14	97%	<0.00001
	monotherapy	1	1.07	0.94-1.21	0.32		
	combination therapy	2	0.45	0.37-0.54	<0.00001	0%	0.34
PD-L1 tumor proportion score 1-49%	Total	2	0.53	0.42-0.69	<0.00001	0%	0.74
	combination therapy	2	0.53	0.42-0.69	<0.00001	0%	0.74
PD-L1 tumor proportion score ≥50%	Total	4	0.50	0.32-0.76	0.001	87%	<0.0001
	monotherapy	2	0.65	0.40-1.04	0.07		
	combination therapy	2	0.37	0.28-0.48	<0.00001	0%	0.93

Table S2 The sensitivity analyses of the studies

Sensitivity analyses	No. of studies	OS HR (95% CI)	*p* value
Total studies	5	0.66 (0.55, 0.79)	p <0.00001
KEYNOTE-021 excluded	4	0.67 (0.55, 0.81)	p <0.0001
KEYNOTE-189 excluded	4	0.74 (0.66, 0.83)	p <0.00001
KEYNOTE-042 excluded	4	0.60 (0.52, 0.69)	p <0.00001
KEYNOTE-407 excluded	4	0.66 (0.52, 0.82)	p =0.0003
KEYNOTE-024 excluded	4	0.66 (0.53, 0.82)	p =0.0002

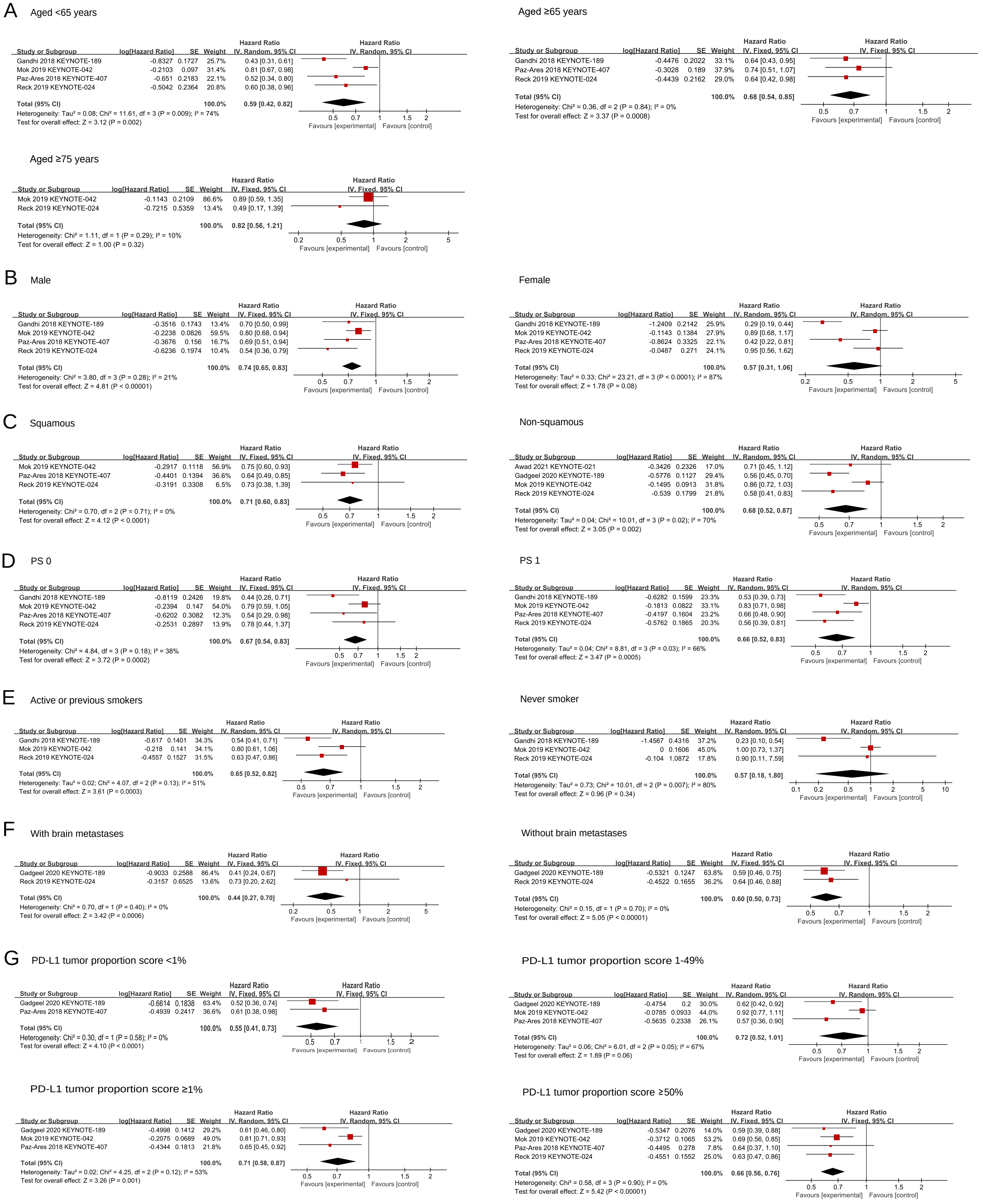

Figure S1: Forest plots of HRs comparing OS between pembrolizumab based therapy and chemotherapy with respect to (A) age group, (B) gender, (C) histomorphological subtypes, (D) PS score, (E) smoking status, (F) brain metastases status, and (G) PD-L1 tumor proportion score.

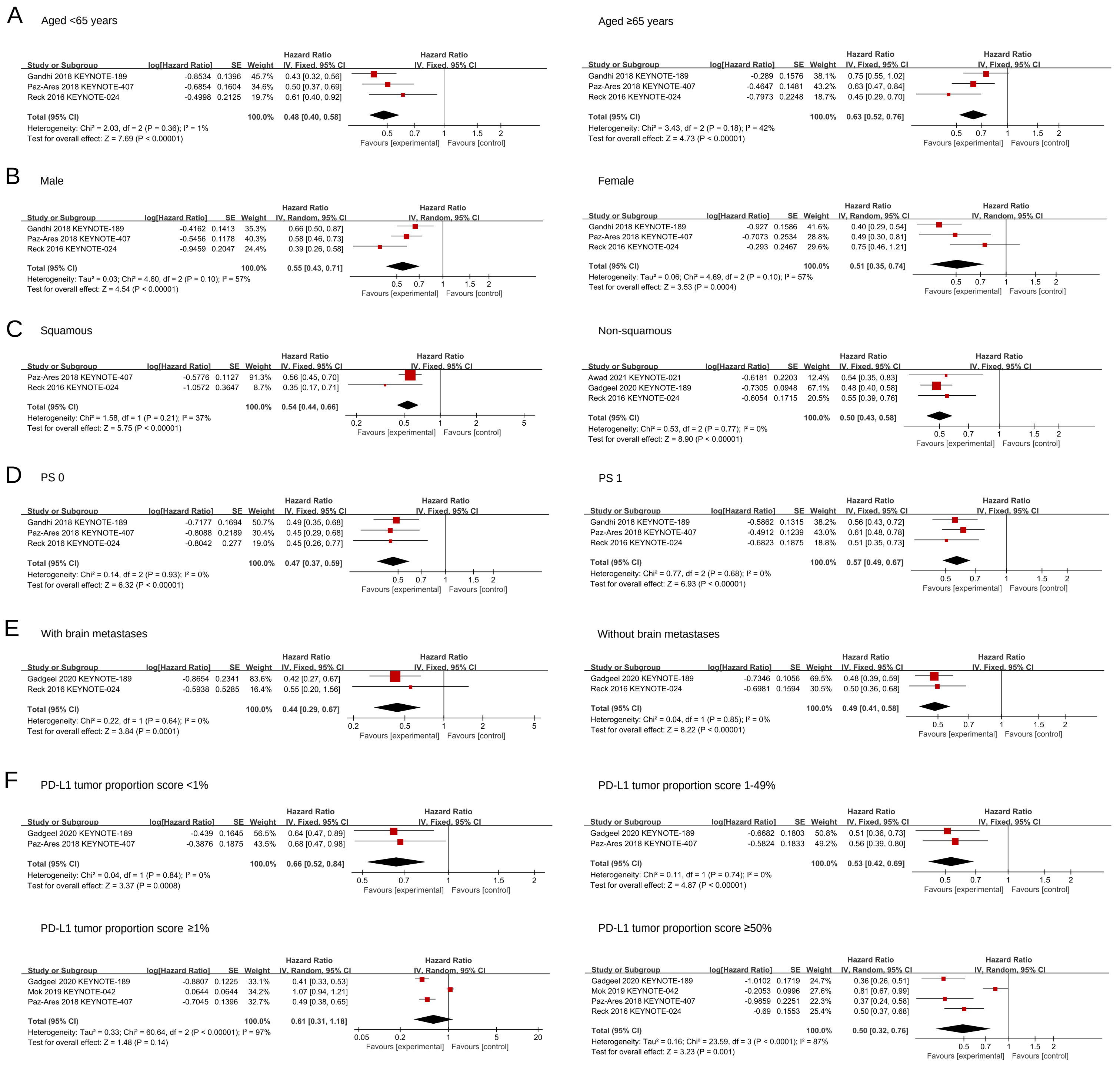

Figure S2: Forest plots of HRs comparing PFS between pembrolizumab based therapy and chemotherapy with respect to (A) age group, (B) gender, (C) histomorphological subtypes, (D) PS score, (E) brain metastases status, (F) PD-L1 tumor proportion score.

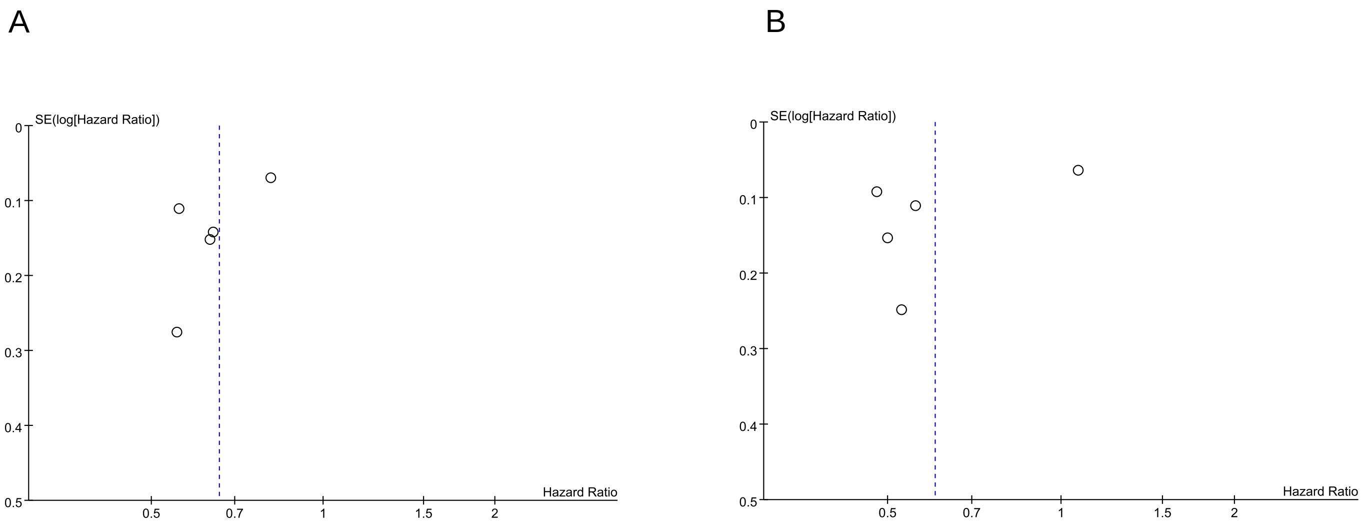

Figure S3: Funnel plots for (A) OS and (B) PFS between between pembrolizumab based therapy and chemotherapy.

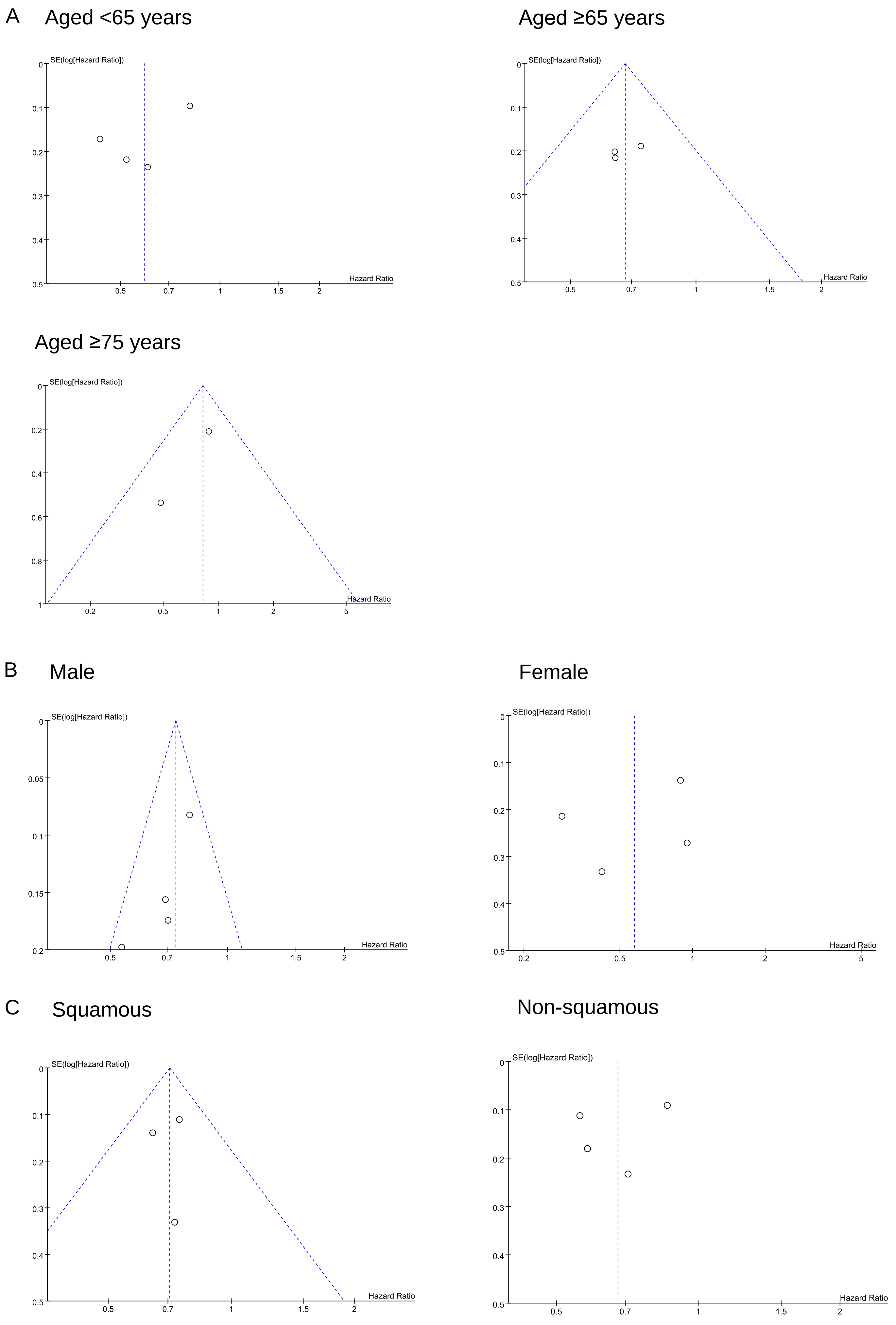
A
Aged <65 years
SE(log[Hazard Ratio])
Hazard Ratio
Aged ≥65 years
SE(log[Hazard Ratio])
Hazard Ratio
Aged ≥75 years
SE(log[Hazard Ratio])
Hazard Ratio
B
Male
SE(log[Hazard Ratio])
Hazard Ratio
Female
SE(log[Hazard Ratio])
Hazard Ratio
C
Squamous
SE(log[Hazard Ratio])
Hazard Ratio
Non-squamous
SE(log[Hazard Ratio])
Hazard Ratio

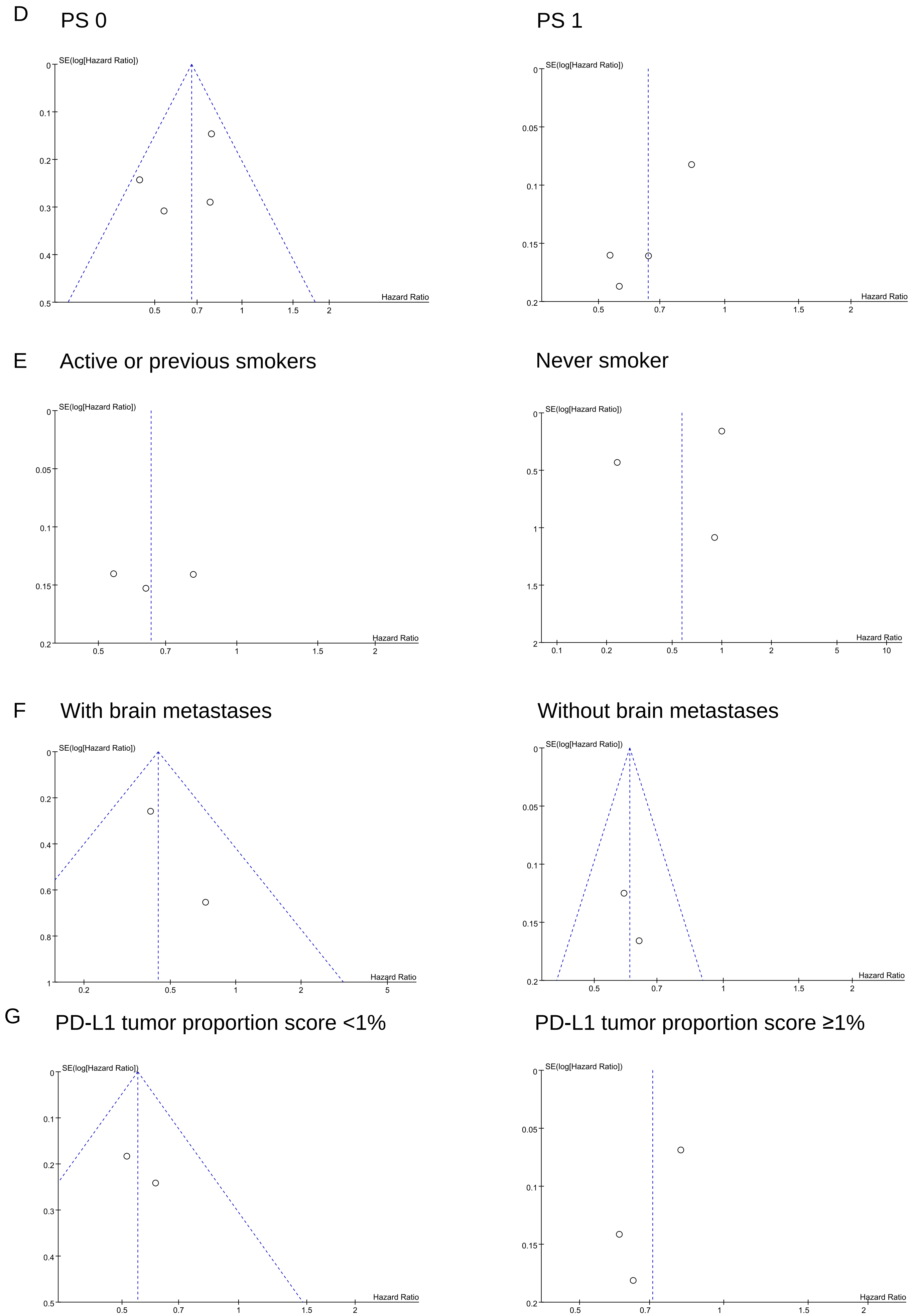
D
PS 0
SE(log[Hazard Ratio])
Hazard Ratio
PS 1
SE(log[Hazard Ratio])
Hazard Ratio
E
Active or previous smokers
SE(log[Hazard Ratio])
Hazard Ratio
Never smoker
SE(log[Hazard Ratio])
Hazard Ratio
F
With brain metastases
SE(log[Hazard Ratio])
Hazard Ratio
Without brain metastases
SE(log[Hazard Ratio])
Hazard Ratio
G
PD-L1 tumor proportion score <1%
SE(log[Hazard Ratio])
Hazard Ratio
PD-L1 tumor proportion score ≥1%
SE(log[Hazard Ratio])
Hazard Ratio

PD-L1 tumor proportion score 1-49%

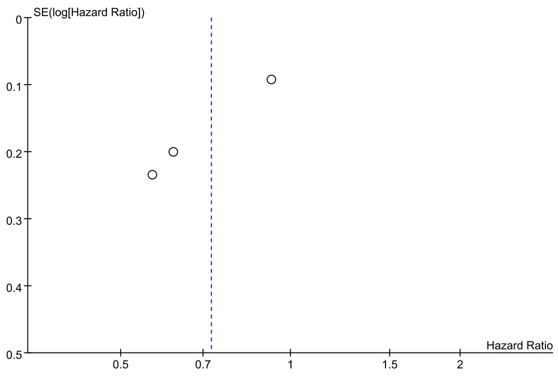

PD-L1 tumor proportion score ≥50%

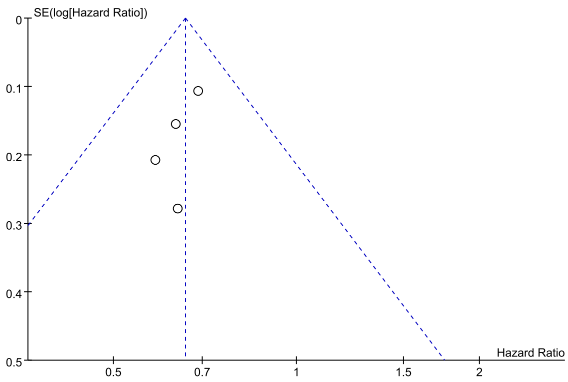

Figure S4: Funnel plots for OS in the subgroup with respect to (A) age group, (B) gender, (C) histomorphological subtypes, (D) PS score, (E) smoking status, (F) brain metastases status, and (G) PD-L1 tumor proportion score.

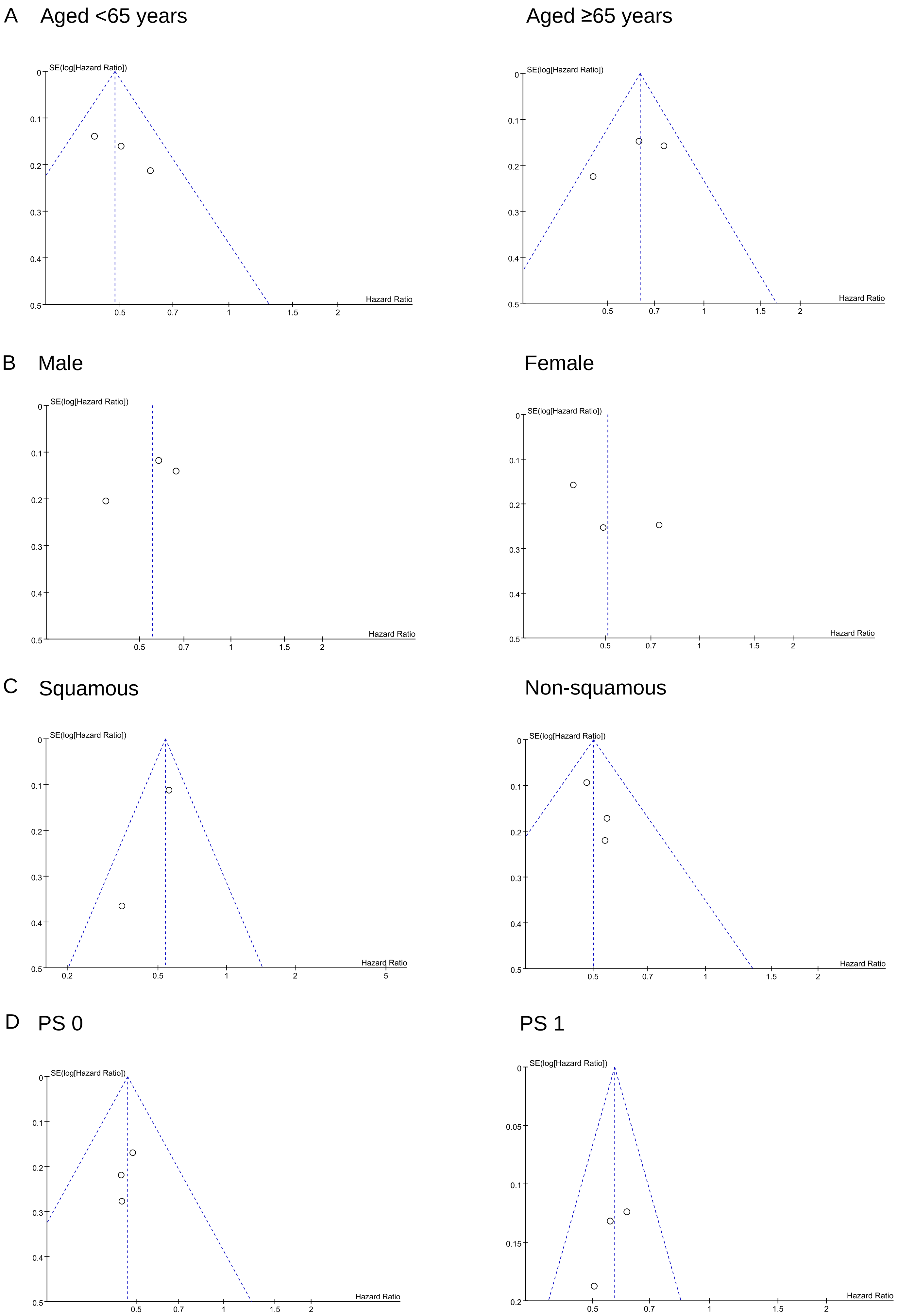
A
Aged <65 years
Aged ≥65 years
B
Male
Female
C
Squamous
Non-squamous
D
PS 0
PS 1
SE(log[Hazard Ratio])
Hazard Ratio

E With brain metastases

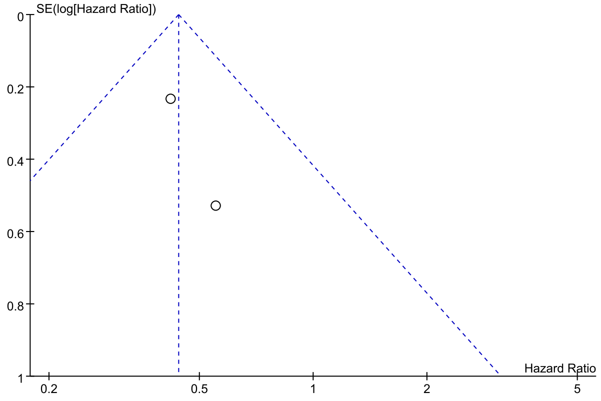

Without brain metastases

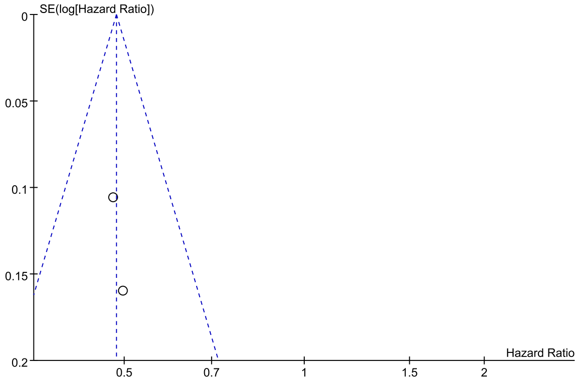

F PD-L1 tumor proportion score <1%

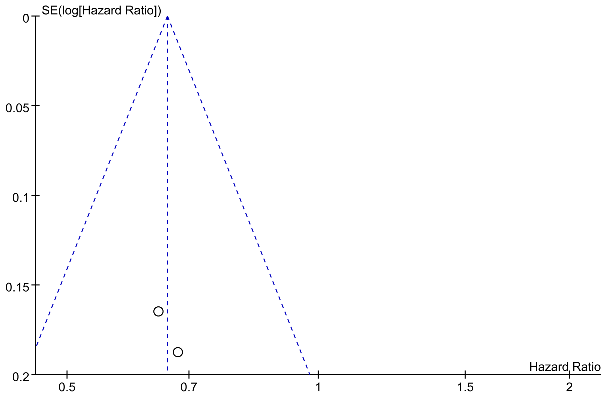

PD-L1 tumor proportion score ≥1%

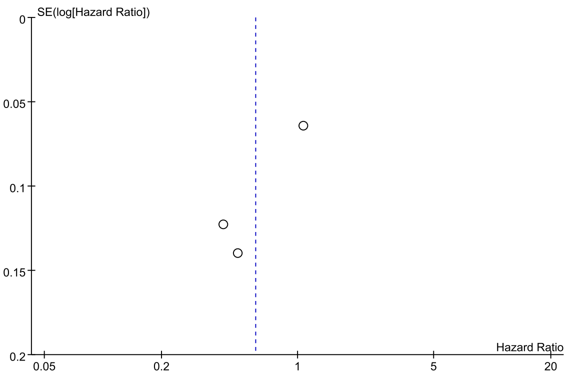

PD-L1 tumor proportion score 1-49%

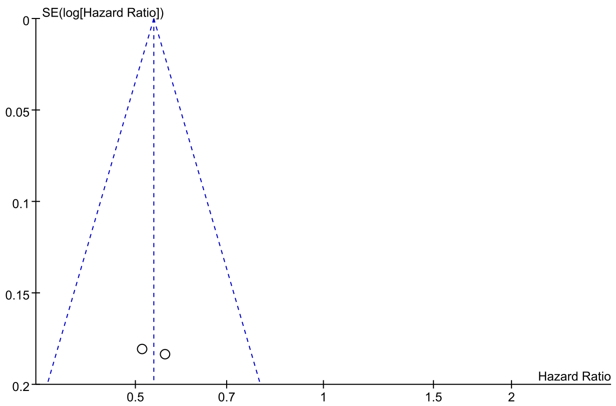

PD-L1 tumor proportion score ≥50%

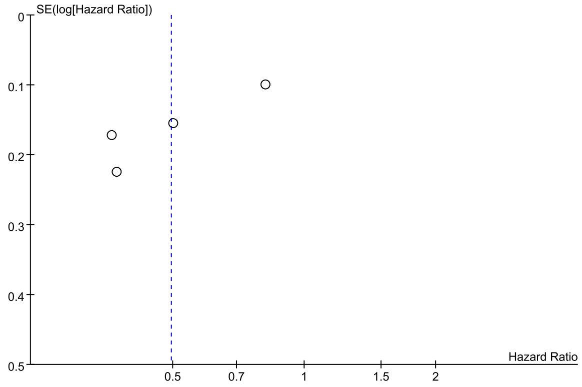

Figure S5: Funnel plots for PFS in the subgroup with respect to (A) age group, (B) gender, (C) histomorphological subtypes, (D) PS score, (E) brain metastases status, and (F) PD-L1 tumor proportion score.
